# Quantitative Synthesis of Factors Associated with COVID-19 Vaccine Acceptance and Vaccine Hesitancy in 185 Countries

**DOI:** 10.3390/vaccines12010034

**Published:** 2023-12-28

**Authors:** Jerome Nyhalah Dinga, Severin Kabakama, Dieudonne Lemuh Njimoh, Julius Ebua Chia, Imran Morhason-Bello, Ivan Lumu

**Affiliations:** 1 Michael Gahnyam Gbeugvat Foundation, Buea P.O. Box 63, Cameroon; 2 Biotechnology Unit, University of Buea, Buea P.O. Box 63, Cameroon; 3 Humanitarian and Public Health Consultant, Mwanza P.O. Box 511, Tanzania; 4 Department of Biochemistry and Molecular Biology, University of Buea, Buea P.O. Box 63, Cameroon; 5 World Health Organization-Regional Office for Africa, Brazaville P.O. Box 06, Congo; 6 College of Medicine, University of Ibadan, Ibadan 200284, Nigeria; 7 Infectious Diseases Institute, Makerere University College of Health Sciences, Kampala P.O. Box 7072, Uganda

**Keywords:** COVID-19, health belief model, vaccine acceptance, vaccine hesitancy, gross domestic product, quantitative synthesis, world bank income level, meta-analysis

## Abstract

Mass vaccination against COVID-19 is the best method to ensure herd immunity in order to curb the effect of the pandemic on the global economy. It is therefore important to assess the determinants of COVID-19 vaccine acceptance and hesitancy on a global scale. Factors were recorded from cross-sectional studies analyzed with *t*-Test, ANOVA, correlation, and meta-regression analyses and synthesized to identify global trends in order to inform policy. We registered the protocol (ID: CRD42022350418) and used standard Cochrane methods and PRISMA guidelines to collect and synthesize cross-sectional articles published between January 2020 and August 2023. A total of 67 articles with 576 studies from 185 countries involving 3081,766 participants were included in this synthesis. Global COVID-19 vaccine acceptance was 65.27% (95% CI; 62.72–67.84%), while global vaccine hesitancy stood at 32.1% (95% CI; 29.05–35.17%). One-Way ANOVA showed that there was no significant difference in the percentage Gross Domestic Product spent on vaccine procurement across the World Bank income levels (*p* < 0.187). There was a significant difference of vaccine acceptance (*p* < 0.001) and vaccine hesitancy (*p* < 0.005) across the different World Bank Income levels. World Bank income level had a strong influence on COVID-19 vaccine acceptance (*p* < 0.0004) and hesitancy (*p* < 0.003) but percentage Gross Domestic Product spent on vaccine procurement did not. There was no correlation between percentage Gross Domestic Product spent on vaccine procurement and COVID-19 vaccine acceptance (r = −0.11, *p* < 0.164) or vaccine hesitancy (r = −0.09, *p* < 0.234). Meta-regression analysis showed that living in an urban setting (OR = 4.83, 95% CI; 0.67–212.8), rural setting (OR = 2.53, 95% CI; 0.29–119.33), older (OR = 1.98, 95% CI; 0.99–4.07), higher education (OR = 1.76, 95% CI; 0.85–3.81), and being a low income earner (OR = 2.85, 95% CI; 0.45–30.63) increased the odds of high COVID-19 vaccine acceptance. Factors that increased the odds of high COVID-19 vaccine hesitancy were no influenza vaccine (OR = 33.06, 95% CI; 5.03–1395.01), mistrust for vaccines (OR = 3.91, 95% CI; 1.92–8.24), complacency (OR = 2.86, 95% CI; 1.02–8.83), pregnancy (OR = 2.3, 95% CI; 0.12–141.76), taking traditional herbs (OR = 2.15, 95% CI; 0.52–10.42), being female (OR = 1.53, 95% CI; 0.78–3.01), and safety concerns (OR = 1.29, 95% CI; 0.67–2.51). We proposed a number of recommendations to increase vaccine acceptance and ensure global herd immunity against COVID-19.

## 1. Introduction

The COVID-19 pandemic has caused significant disruption in healthcare services and presented substantial challenges to governments [1]. This led to the implementation of several measures such as improved hand hygiene, use of personal protective equipment, rapid testing and vaccination, and social distancing to curtail the spread of the different variants of the SARS-CoV-2 virus [2,3,4,5,6,7,8]. However, there is a global consensus that vaccination is the most effective public health intervention that can be used to curb the spread of the disease [9,10,11,12,13,14]. This background notion led to increased efforts worldwide to rapidly develop COVID-19 vaccines [15,16]. This was due to the fact that leading scientists around the world had access to huge amounts of funding provided by funding agencies [16,17,18]. As of 28 November 2023, WHO granted emergency use listing for 12 vaccines [19] that have been pre-qualified by WHO for massive administration around the world. Another benefit of vaccination is that it will enhance the health status of people, leading to a better life quality and contribute to economic development [20,21,22]. Hence, vaccination can contribute to 14 of the 17 Sustainable Development Goals set by the World Health Organization [23].

One of the factors that ensures the success of vaccination programs is sustained financing from the Gross Domestic Product (GDP), which requires long term commitment and consistent resources [24]. Given the differences in the economy of countries around the world, it is obvious that not all countries were able to purchase the necessary vaccines with the same ease [25,26]. In fact, vaccine procurement platforms were created to assist low income and lower-middle income countries to procure the much-needed vaccines against COVID-19 to immunize their population [27]. These include COVAX, UNICEF Supply, UNICEF Vaccine Independent Initiative [28], and Economic Community of West African States revolving fund [29]. Despite the availability of these vaccines, COVID-19 continues to spread, and it is therefore imperative to look at the reasons for this low vaccination coverage from a systemic global perspective.

Barriers to vaccine uptake are multi-dimensional, including demographical (age, sex, race, ethnicity, and education, among others), psychosocial (personality, social class, and peer and reference group pressure, among others), and structural (cost, convenience, supply chain issues) factors [30]. The World Health Organization (WHO) defines vaccine hesitancy (VH) as “delay in acceptance or refusal of vaccines despite availability of vaccine services” [31]. Among other factors, vaccine hesitancy plays the principle role in low vaccine acceptance (VA) with the most common determinants being low health literacy, context-specific safety-related concerns, and mistrust [32,33]. Additionally, primary healthcare workers remain an important component of the taskforce to tackle VH, so, lack of training or low confidence in this group of persons will definitely reduce the potential to overcome public VH [9,34,35,36,37].

Even though global rates of COVID-19 vaccination are gradually improving, but in an uneven manner, there is evidence that suggest antibody response to vaccination wanes rapidly, necessitating the administration of booster doses to achieve adequate levels of protection [38,39,40,41]. Hence, there is a need to increase VA and reduce VH in order to establish herd immunity [42,43,44,45,46,47,48,49,50].

VH remains a complex phenomenon, with more than 70 factors identified that influence it, many of which are context-specific and time-specific [51,52,53,54,55,56]. It is therefore expected that factors that influence hesitancy to accept the first COVID-19 dose will also affect hesitancy to the second or booster doses. Acceptance is also affected by the inability of current vaccines to stop the infection of new circulating variants [57,58,59,60,61,62]. Here, we carried out a global quantitative synthesis to identify determinants of COVID-19 vaccine acceptance and hesitancy identified in 185 countries.

## 2. Materials and Methods

### 2.1. Study Design

A systematic review and meta-analysis of studies were conducted to assess the factors associated with global COVID-19 VA and VH. The Preferred Reporting Items for Systematic Reviews and Meta-Analyses (PRISMA) guidelines [63] were followed to review articles of the included studies. Ethics review and approval are not required for analyses of published data.

### 2.2. Eligibility Criteria

The criteria for inclusion include cross-sectional studies that report the proportion of COVID-19 VA and/or COVID-19 VH. The study must include statistical analysis to identify the associated factors for COVID-19 vaccine acceptance and/or hesitancy. Studies with cross-sectional design published in English from January 2020 to August 2023 were included in the synthesis. The study focused only on adults and parental vaccine acceptance and hesitancy was excluded. Only countries that had a study of either VA or VH or both were included in the synthesis. Countries with neither VA nor VH studies were excluded. Case series/reports, cohort designs, case-control, conference papers, proceedings, articles available only in abstract form, editorial reviews, letters of communications, commentaries, webpages, and qualitative studies were also excluded. Articles in languages other than English were not included in this study.

### 2.3. Search Strategy

The search was conducted using the generic free-text search terms “COVID-19 vaccine acceptance country name = e.g., Afghanistan OR Albania OR Algeria, … OR Zimbabwe” OR “COVID-19 vaccine hesitancy country name = e.g., Afghanistan OR Albania OR Algeria, … Zimbabwe”. All studies published from 2020 to August 2023 were retrieved to assess their eligibility for inclusion in this study. The search was restricted to full-text only and English language articles in online databases MEDLINE and Google Scholar. To find additional potentially eligible studies, reference lists of included citations were cross-checked.

### 2.4. Selection Process

**Figure 1:**
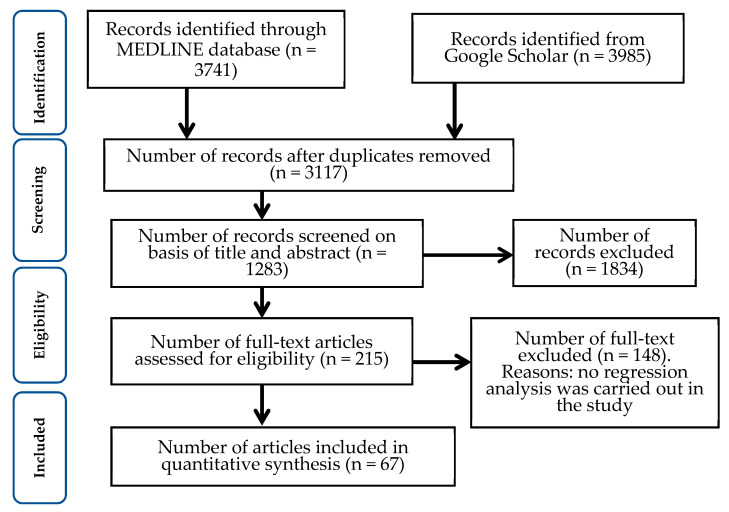
PRISMA flow diagram of the literature search. From a total of 7726 studies identified, we removed 4609 duplicates; we screened 1283 studies for eligibility and excluded 148 studies not reporting regression analysis to identify VA or VH factors. Therefore, we finally included a total of 67 eligible studies for this quantitative synthesis.

All records identified by our search strategy were exported to Zotero software version 6.0.30. Duplicate articles were removed from the list. Two independent reviewers screened the titles and abstracts of the identified articles for inclusion in the synthesis. A third reviewer checked and resolved any event of a conflict between the two independent reviewers. The search method was presented in the PRISMA flow chart showing the included studies and excluded with reasons for exclusion (Figure 1).

### 2.5. Secondary Data Analysis

Countries were divided into low-income countries (LIC), lower-middle income countries (LMIC), upper-middle income countries (UMIC), and high-income countries (HIC) based on the World Bank (WB) income group categorization [64], and used for secondary quantitative and meta-regression analysis.

Percentage GDP spent on vaccine procurement were obtained directly from internet search or calculated from GDP, expenditures on vaccines per capita, percentage health budget for vaccines, percentage of GDP for health budget [65], and the country population [66].

If there are three studies identifying three different VA rates for a particular country, the average value of the three is recorded and used for analysis. Individual studies from each country were counted as such, e.g., five studies from Ethiopia are counted as five. Factors for COVID-19 VA and VH were collected separately and analyzed independently. Factors were counted as one per country no matter the number of studies identifying that factor for that country, e.g., if five studies identify that being female is a factor for VH in Egypt, female is recorded as one VH factor for Egypt. Identified factors were grouped using the Health Belief Model [67] (demographic, psychosocial, and structural independent variables) (Supplementary Material; Tables S1 and S2) for further analysis in this study.

### 2.6. Data Collection Process and Data Items

The data were extracted into Microsoft Excel (Microsoft Office Professional 2010). R Programming was used for statistical analysis and generating plots.

### 2.7. Reporting Bias Assessment

The risk of bias was assessed by six criteria [68]: (1) cross-sectional study, (2) appropriateness of study participants sampled, (3) adequate of sample size, (4) description of study subjects and the setting, (5) sample size justification or power description, (6) appropriateness of statistical analysis.

### 2.8. Protocol and Registration

The study protocol was registered in the PROSPERO, International prospective register of systematic reviews under decree code of CRD42022350444.

## 3. Results

### 3.1. Demographic Analysis

**Figure 2:**
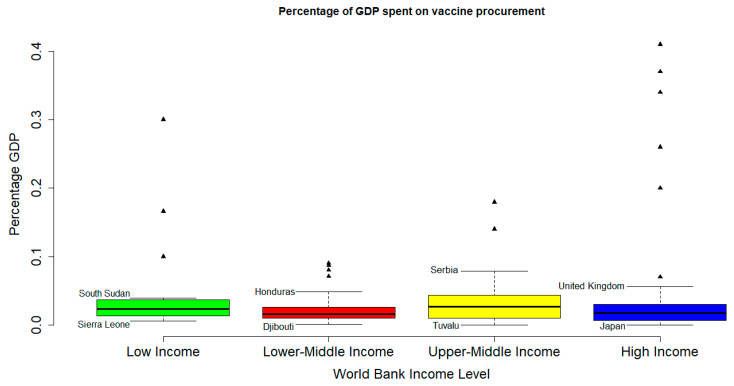
Percentage GDP spent on vaccine procurement per World Bank income level. Countries indicated are, respectively, countries with the highest and lowest percentage GDP spent on vaccine procurement for each WB income level. Triangular marks are outliers. The Interquartile range (IQR) criterion indicates that all data points above _q0.75_ + 1.5 (IQR) or below _q0.25_ − 1.5 (IQR) (where _q0.25_ and _q0.75_ correspond to first and third quartile respectively, and IQR is the difference between the third and first quartile) are considered as potential outliers by R. That is, all observations outside of the following interval were considered as outliers: I = [_q0.25_ − 1.5 (IQR); _q0.75_ + 1.5 (IQR)].

Sixty-seven (67) records with five hundred and seventy-seven studies involving one hundred and eighty-five (185) countries were included in this study; twenty-three LIC, fifty-four LMIC, forty-nine UMIC, and fifty-nine LIC. These studies involved 3,081,766 participants. There was no significant difference in the percentage of GDP spent on vaccine procurement in the different WB income levels (*p* < 0.187) (Figure 2). No records of COVID-19 VA or VH was found for Cuba, Equatorial Guinea, Eritrea, Iceland, North Korea, Moldova, Monaco, San Marino, Turkmenistan, and Madagascar. Table 1 shows the demographic characteristics of the study.

Table 2 shows the list of records as identified by the income level of the country in which the cross-sectional studies were carried out.

### 3.2. COVID-19 Vaccine Acceptance

**Figure 3:**
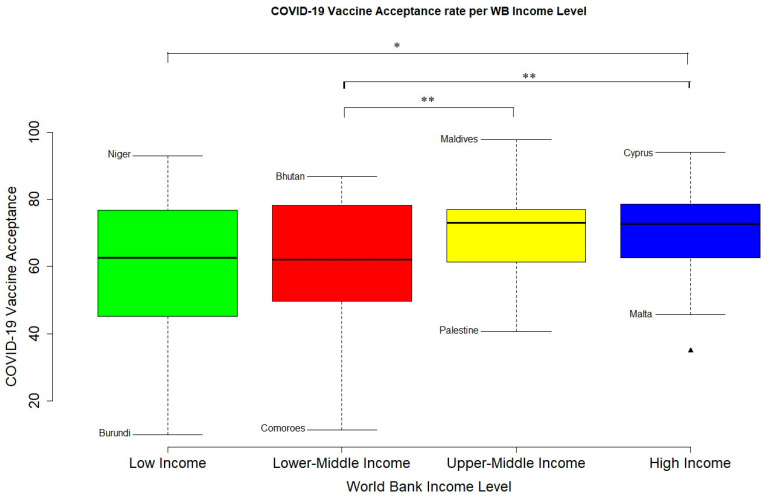
COVID-19 * vaccine acceptance rate per World Bank Income Level. Countries indicated are, respectively, countries with the highest and lowest VA rate for each WB income level. Triangular mark is an outlier. The IQR criterion indicates that all data points above _q0.75_ + 1.5 (IQR) or below _q0.25_ − 1.5 (IQR) (where _q0.25_ and _q0.75_ correspond to first and third quartile respectively, and IQR is the difference between the third and first quartile) are considered as potential outliers by R. That is, observation outside of the following interval was considered an outlier: I = [_q0.25_ − 1.5 (IQR); _q0.75_ + 1.5 (IQR)]. * indicates *p*-value was less than 0.05 and ** indicates that *p*-value was less than 0.01.

**Figure 4:**
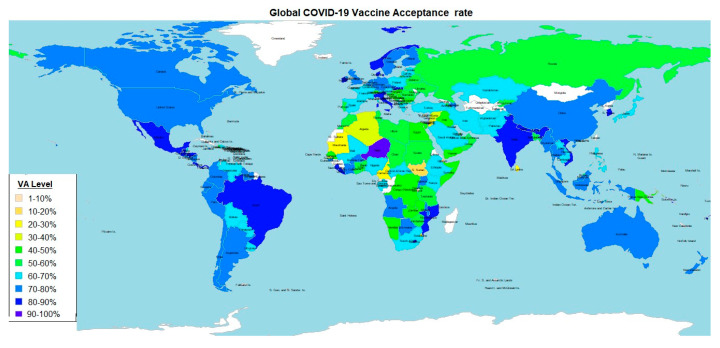
**Worldmap showing distribution of VA rates amongst the nations of the world using the rworldmap package in R** [128]. HIC and UMIC have higher VA than countries in LMIC and LIC. Visual inspection of a world map of COVID-19 VA shows that countries with high VA were mostly found in the Americas, Asia, and Europe while countries with low VA were mostly located in Africa and the Middle East. Countries with no recorded data are in white.

Global COVID-19 vaccine acceptance was 65.27% (95% CI; 62.72–67.84%). There was a significant difference vaccine acceptance across the different World Bank Income levels (*p* < 0.001) (Table 1) (Figure 3 and Figure 4). Two-sample *t*-Test performed between each two groups showed that HIC had a significantly higher VA than LMIC (*p* < 0.002) and LIC (*p* < 0.04) but not UMIC (*p* < 0.67). UMIC had a significantly higher VA than LMIC (*p* < 0.006) but not more than LIC (*p* < 0.07) and HIC (*p* < 0.67) (Figure 3 and Figure 4). Analysis of Variance showed that WB income level was associated to COVID-19 vaccine acceptance (*p* < 0.0004) but percentage GDP spent on vaccine procurement did not (*p* < 0.426). Pearson’s product-moment correlation coefficient test showed that percentage GDP spent on vaccine procurement did not correlate with COVID-19 vaccine acceptance (r = −0.11, *p* < 0.164). Visual inspection of a world map of COVID-19 VA shows that countries with high VA were mostly found in the Americas, Asia, and Europe while countries with low VA were mostly located in Africa and the Middle East (Figure 4).

**Figure 5:**
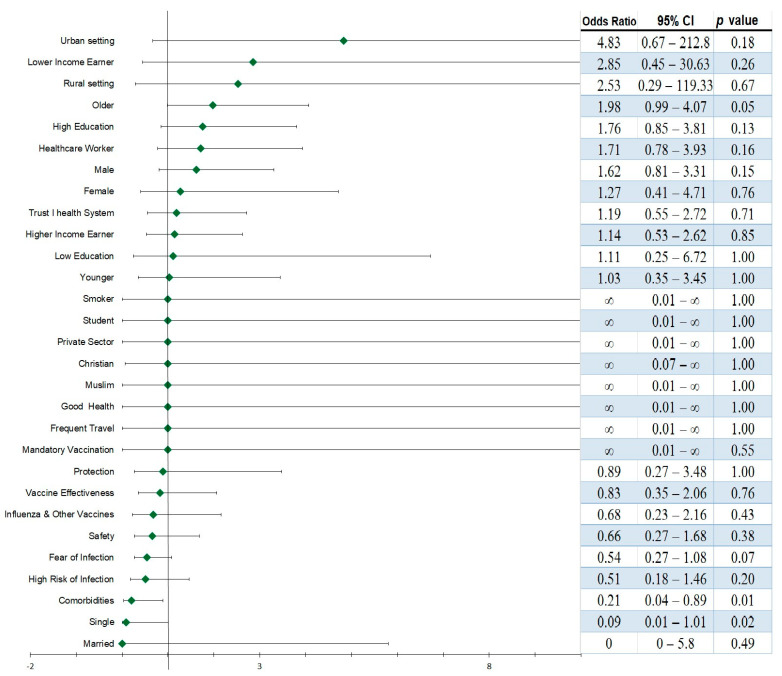
**Meta-regression analysis to identify factors that influence COVID-19 vaccine acceptance in 185 countries.** Factors that were identified as having a strong effect on VA in each study for each country were recorded, grouped according to the Health Belief Model, and analyzed against ≥VA 60% as the outcome. VA of 60% and above was considered high VA and used for the synthesis. A factor with OR above 1 was considered a factor that increased the odds of high VA. *p* < 0.05 was considered statistically significant.

Meta-regression analysis was performed to identify factors that strongly increase the chances of COVID-19 VA. Factors that were identified as having a strong effect on VA in each study for each country were recorded, grouped according to the Health Belief Model, and analyzed against the VA rates of 60% and above as the outcome (which was considered high VA for this synthesis). Logistic regression was used to calculate the possibility of a binary outcome (high VA (≥60%) and low VA (<60%)) when exposed to each of the independent variables (factors) being studied (Figure 5). Living in an urban setting increased the odds of high VA by 4.83 (OR = 4.83, 95% CI; 0.67–212.8), living in a rural setting increased the odds of high VA by 2.53 (OR = 2.53, 95% CI; 0.29–119.33), and older persons by 1.98 (OR = 1.98, 95% CI; 0.99–4.07). Other factors that increased the odds of high VA were having attained higher education (OR = 1.76, 95% CI; 0.85–3.81) and being a low-income earner (OR = 2.85, 95% CI; 0.45–30.63). However, these increased odds for high VA was statistically significant only for older persons (*p* < 0.05) (Figure 5).

Table 3 shows the top five factors that have been identified in countries in the different WB income levels as being associated with COVID-19 vaccine acceptance. Fear of infection with COVID-19 appeared to be the most frequent reason of accepting to take a COVID-19 vaccine (Table 3).

### 3.3. COVID-19 Vaccine Hesitancy

**Figure 6:**
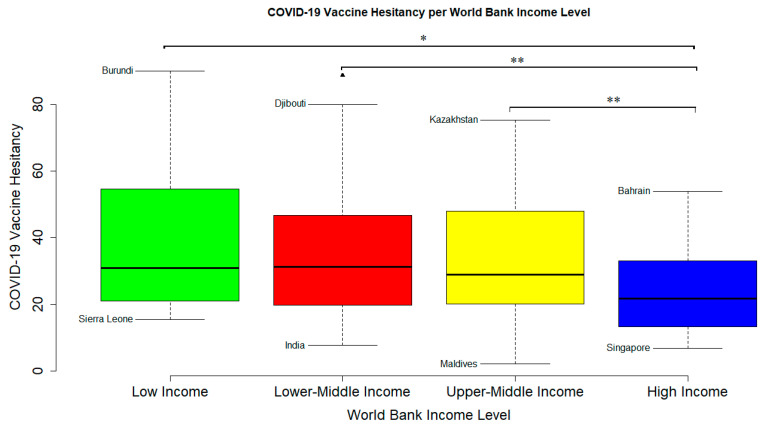
**COVID-19 Vaccine Hesitancy level across different countries of the different World Bank Income levels**. Countries indicated are, respectively, countries with the highest and lowest VH rate for each WB income level. Triangular mark is an outlier. The IQR criterion indicates that all data points above _q0.75_ + 1.5 (IQR) or below _q0.25_ − 1.5 (IQR) (where _q0.25_ and _q0.75_ correspond to first and third quartile, respectively, and IQR is the difference between the third and first quartile) are considered as potential outliers by R. That is, any observation outside of the following interval was considered an outlier: I = [_q0.25_ – 1.5 (IQR); _q0.75_ + 1.5 (IQR)]. * indicates *p*-value was less than 0.05 and ** indicates that *p*-value was less than 0.01.

**Figure 7:**
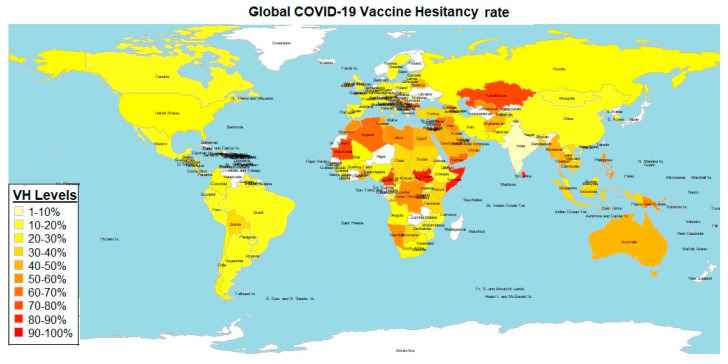
**Worldmap showing VH rates around the world was drawn using the rworldmap package in R programming** [128]. Countries with high VH were mostly located in Africa and the Middle East while countries with low VH were mostly located in the Americas, Asia, and Europe. Countries with no recorded data are in white.

We calculated a global COVID-19 vaccine hesitancy rate of 32.11% (95% CI; 29.05–35.17%) (Table 1). As shown in Figure 6, there was a significant difference of the level of COVID-19 vaccine hesitancy across the different WB Income levels (*p* < 0.005) (Figure 6). Two-sample T-Test performed between each two groups showed that LIC had a significantly higher VH than HIC (*p* < 0.013) but not more than LMIC (*p* < 0.605) and UMIC (*p* < 0.281). LMIC had a significantly higher VH than HIC (*p* < 0.002) but not more than UMIC (*p* < 0.424). UMIC showed a significantly higher VH than HIC (*p* < 0.008) (Figure 6 and Figure 7). Two-Way ANOVA analysis showed that WB income level was associated with VH (*p* < 0.002) but percentage GDP spent on vaccine procurement did not (*p* < 0.599). Also, Pearson’s product-moment correlation coefficient showed that VH is not correlated with percentage GDP spent on vaccine procurement (r = −0.09, *p* < 0.234). A world map of VH showed that countries with high VH were mostly located in Africa and the Middle East while countries with low VH were mostly located in the Americas, Asia, and Europe (Figure 7).

**Figure 8:**
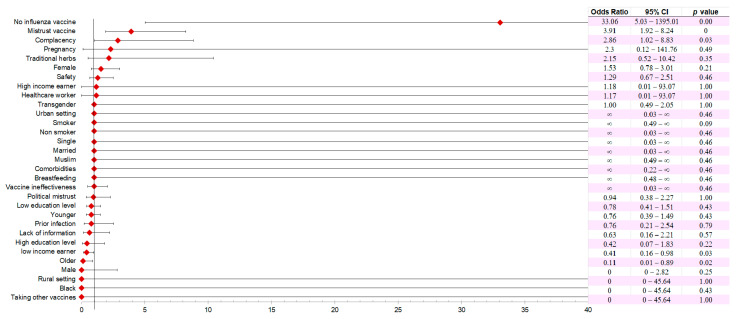
Meta-regression analysis to identify factors that significantly increase the chances of being hesitant to a COVID-19 at national levels. VH of 30% and above was considered high VH and used for the synthesis. Factors that were identified as having a strong effect on VH in each study for each country were recorded, grouped according to the Health Belief Model, and analyzed against ≥VH 30% as the outcome. VH of 30% and above was considered high VH and used for the synthesis. A factor with OR above 1 was considered a factor that increased the odds of high VH. *p* < 0.05 was considered statistically significant.

For meta-regression analysis, factors identified as having a strong effect on VH in each study for each country were recorded, grouped according to the Health Belief Model, and analyzed against VA rates of 30% and above as the outcome. COVID-19 vaccine hesitancy of 30% and above was considered high VH for the purpose of this synthesis and associated factors identified using meta-regression analysis. It was observed that not taking the influenza vaccine increased the odds of high VH by 33.06 (OR = 33.06, 95% CI; 5.03–1395.01), mistrust for vaccines by 3.91 (OR = 3.91, 95% CI; 1.92–8.24) and complacency by 2.86 (OR = 2.86, 95% CI; 1.02–8.83). Other factors that increased the odds of high VH were pregnancy (OR = 2.3, 95% CI; 0.12–141.76), taking traditional herbs (OR = 2.15, 95% CI; 0.52–10.42), being female (OR = 1.53, 95% CI; 0.78–3.01), and safety concerns (OR = 1.29, 95% CI; 0.67–2.51). However, these increased odds for high VH were statistically significant only for not taking the influenza vaccine (*p* < 0.000), complacency (*p* < 0.03), and mistrust of the vaccine (*p* < 0.000) (Figure 8).

The top five factors that commonly affected VH in the different WB income levels are shown in Table 4. We counted the number of countries in which a factor was identified for each WB income level. This was presented as the number of countries and the percentage number of countries for that WB income level (Table 4). Being female occurred most frequently as being a factor associated with vaccine hesitancy (Table 4).

## 4. Discussion

Vaccination remains the most effective intervention that can help humanity to overcome the COVID-19 pandemic through herd immunity in the communities. The effectiveness of these vaccines depends on the acceptance and uptake by the population. In this quantitative synthesis, the average COVID-19 vaccine acceptance from 577 studies involving 185 countries and 3,081,766 participants was 65.27% (95% CI; 62.72–67.84%, *p* < 0.000). This finding was comparable to other studies that showed a global vaccine acceptance rate of 63.1% [129], 64.9% (95% CI of 60.5 to 69.0%) [130], but was lower when compared to other multi-country studies carried out by others; 67.8% by Wang et al. [131], 69% recorded by Noushad et al. [132], 73.3% recorded by Terry et al. [133], 75.2% by Lazarus et al. [134], 80.3% by Solís Arce et al. [90], and 88.8% by Bono et al. [135]. However, the global VA recorded in this synthesis was higher than the 56% VA reported by Mekonnen and Mingistu [136] and the 61% reported by Norhayati et al. [137]. This could be due to the fact that not all studies included in this synthesis were analyzed in the other studies. Living in an urban setting, rural setting, older persons, higher education, and being a low-income earner were identified in this synthesis as being associated with COVID-19 VA. These were similar to the factors identified by other studies [90,132,138,139,140,141]. However, a different set of factors associated with VA, including history of chronic disease, good knowledge, positive attitude, good COVID-19 preventive practice, and high perceived seriousness of COVID-19, were identified by another meta-analysis [136]. These differences in factors identified could be due to the fact that they change with place, time, and social class and could be cultural, geographical, and context-specific [97,98,139].

Among the factors that were identified in this synthesis that can increase the odds of high VA, the only factor that increased that odds in a statistically significant manner was being an older person. This confirms that older person is a factor associated with VA as identified by other studies [142,143,144,145,146]. This is probably because older persons are more prone to other diseases, leading to comorbidities due to the aging immune system [147,148,149,150,151]. This also makes them more vulnerable to COVID-19 infection with much more severe impact compared to younger persons, for example [151,152,153,154].

Looking at the regions of the world according to the World Bank income classification levels, LIC and LMIC had a lower VA compared to UMIC and HIC. This finding is similar to a study by Qunaibi et al., which showed low VA in LMIC [86]. This may be because low mortality in LIC and LMIC [155] led to complacency, hence reducing VA.

We observed a global COVID-19 vaccine hesitancy prevalence of 32.11%. This was lower compared to other multi-country/meta-analysis studies, which recorded vaccine hesitancy of 38.2% [123], 42.3% [156], and 46% [157] but higher than the VH of 12.1% [158], 21% [159], 26.5% [160], and 30.5% observed by Gulle et al. [161]. These differences could be because not all the studies analyzed in this synthesis were analyzed by the other studies [162]. The predictors of COVID-19 vaccine hesitancy in the study by Kigongo et al. were perceived low risk of COVID-19 infection, vaccine side effects, and negative beliefs towards vaccine [157], whereas in this synthesis, no influenza vaccine, mistrust of vaccine, complacency, pregnancy, being female, and safety concerns were predictor of VH. However, factors similar to the ones identified in this synthesis were also identified in other studies [163,164,165,166]. These discrepancies may be because factors change with time, place, and culture as well as being psychologically- and context-specific [167].

Mistrust of government institutions, public health institutions, scientists, and vaccines have been identified as playing a role in discouraging people from taking the COVID-19 vaccine. This study and others confirmed that political mistrust and mistrust in vaccines, scientists, and public health institutions continued to play a role in increasing COVID-19 vaccine hesitancy [97,103,168,169,170,171,172,173,174,175]. Politicization of vaccines for political gains has been recorded and well exploited. This is due to the inherent nature of science itself, which has to do with the uncertainty of the field and the fact that questioning existing findings is part of the research process [168,176,177,178]. There is, therefore, a need to depoliticize outreach programs by involving health officials that have proven record of being apolitical [168], as well as the use of physicians as most families turn to trusted physicians that have once attended to them successfully [170].

We observed an average hesitancy rate of 44.09% across low income and lower-middle income countries. This was lower when compared to another study that measured hesitancy in LIC and LMIC and observed that more than half the study population were hesitant [74]. Our findings showed that VH was higher in LIC and LMIC than in UMIC and HIC. This was contrary to the study by Cata-Preta et al., which stated that VH was higher in rich countries than in poor countries due to the emergence of VH [179].

Several studies have looked at the impact of GDP on COVID-19 vaccination uptake [180,181,182,183] but none have looked at the impact of GDP on vaccine acceptance or hesitancy. Here we present the first study, to the best of our knowledge, that showed that WB income level was associated with VA and VH, but that percentage GDP spent on vaccine procurement was not associated with VA and VH. This may be because affluent countries have well developed health systems compared to those of developing countries [180]. Hence, people in rich countries trust their health system and will easily accept the vaccine whereas the health system in poor countries is not reliable, leading to mistrust, low VA, and high VH [184,185,186,187].

With the many factors that affect the acceptability and rejection of a COVID-19 vaccine, it is imperative that accurate and up-to-date information is made available to all countries to guide the international community to understand the intricacies of vaccine acceptance and hesitancy and shed light on the blind spots essential for achieving global herd immunity. The present quantitative synthesis sheds more light on the factors that influence vaccine acceptance and hesitancy according to World Bank income classification level and globally.

Looking at all the factors identified through this study and others, we believe if the right information is passed through to everyone, then hesitancy will be reduced to bare minimum. However, approach is very important as information provision alone is unlikely to change attitudes, but broad communication strategies can raise awareness and emphasize shared values and social norms. Governments’ policies should involve communication strategies that have a clear plan of action. Drawing on humor or emotion may increase engagement [188,189]. It is essential that everyone in the community should be involved and be made to “own” the communication campaigns by featuring the voices and stories from diverse people across the community. Implementing a health education plan to reduce pandemic fatigue [190] and taking the concerns of those who have recovered from the disease would be one way to ensure the increase in COVID-19 vaccine acceptance. There may be also a need for medical health personnel to “teach” medicine to the population [191]. This necessitates the development of a comprehensive multidisciplinary and interdisciplinary lines of action to improve both local and international public health policies [192].

## Figures and Tables

**Table 1:** Demographic analysis of the study.

		LIC	LMIC	UMIC	HIC	Total
1.	No. of studies	82	163	142	190	**577**
2.	No. of participants	101,106	1,030,289	1,156,455	793,376	**3,081,766**
3.	Average COVID-19 VA (%, 95% CI)	59.19 (49.31–69.07)	59.13 (53.38–64.89)	68.79 (64.99–72.59)	68.89 (66.42–73.35)	**65.27 (** **62.72%–67.84)**
4.	Average COVID-19 VH (%, 95% CI)	39.32 (28.09–50.55)	36.05 (29.65–42.45)	32.75 (27.52–37.98)	23.96 (20.03–27.89)	**32.11 (29.05–35.17)**
5.	Average Percentage GDP spent on vaccine procurement (%, 95% CI)	0.0461 (0.0172–0.0752)	0.0222 (0.0165–0.0279)	0.0329 (0.0231–0.0427)	0.0447 (0.0202–0.0692)	**0.0350 (0.0262–0.0438)**

**Table 2:** Studies included in this synthesis based on the World Bank Income level of the study site.

	Income Category	Studies on COVID-19 VA and VH Included in the Synthesis
1.	Low Income Countries	Azimi et al., 2023, Abay et al., 2022; Ackah et al., 2022; Africa CDC, 2021; Ahmed et al., 2021; Dayton et al., 2022; De Figueiredo et al., 2023; De Sousa et al., 2021; Dereje et al., 2022; Ditekemena et al., 2021; Echoru et al., 2021; Kanyanda et al., 2021; Mebarki et al., 2023; Mesele, 2021; Mohammed et al., 2021; Mose and Yeshaneh, 2021; Patwary, Bardhan, et al., 2022; Qunaibi et al., 2021; Riad et al., 2021; Rice et al., 2022; Sallam et al., 2022; Solís Arce et al., 2021; Takoudjou Dzomo et al., 2023 [69,70,71,72,73,74,75,76,77,78,79,80,81,82,83,84,85,86,87,88,89,90,91]
2.	Lower-middle Income Countries	Ackah et al., 2022; Africa CDC, 2021; Ajonina-Ekoti et al., 2022; Ali and Hossain, 2021; Amour et al., 2023; Avahoundje et al., 2022; Ba et al., 2022; Dayton et al., 2022; De Figueiredo et al., 2023; De Sousa et al., 2021; Dinga et al., 2021, 2022; M. B. Hossain et al., 2021; Md. S. Hossain et al., 2022; Kacimi et al., 2022; Kanyanda et al., 2021; Lamptey et al., 2021; Lazarus et al., 2023; Lounis et al., 2022; Marzo et al., 2022; Mebarki et al., 2023; Mudenda et al., 2022; Padonou et al., 2023; Patwary, Bardhan, et al., 2022; Puertas et al., 2022; Qunaibi et al., 2021; Riad et al., 2021; Sallam et al., 2022; Solís Arce et al., 2021 [71,72,74,75,76,80,81,85,86,87,89,90,92,93,94,95,96,97,98,99,100,101,102,103,104,105,106,107,108]
3.	Upper-middle Income Countries	Ackah et al., 2022; Daşıkan et al., 2023; Dayton et al., 2022; De Figueiredo et al., 2023; De Sousa et al., 2021; Doran et al., 2022; Gentile et al., 2021; Jorgensen et al., 2023; Lazarus et al., 2023; Marzo et al., 2022; Matovic Miljanovic et al., 2022; Patwary, Bardhan, et al., 2022; Puertas et al., 2022; Qunaibi et al., 2021; Riad et al., 2021; Sallam et al., 2022; Šljivo et al., 2021; Solís Arce et al., 2021 [71,74,75,76,85,86,87,89,90,103,105,108,109,110,111,112,113,114]
4.	High Income Countries	Cuschieri & Grech, 2022; De Figueiredo et al., 2023; De Sousa et al., 2021; Di Valerio et al., 2022; Gagneux-Brunon et al., 2021; Galanis et al., 2022; Kelly et al., 2021; King et al., 2021; Lazarus et al., 2023; Lindholt et al., 2021; Murphy et al., 2021; Patwary, Alam, et al., 2022; Patwary, Bardhan, et al., 2022; Puertas et al., 2022; Qunaibi et al., 2021; Riad et al., 2021; Robertson et al., 2021; Sallam et al., 2022; Solís Arce et al., 2021; Stamm et al., 2022; UNICEF, 2022; Verger et al., 2021 [75,76,85,86,87,89,90,103,108,115,116,117,118,119,120,121,122,123,124,125,126,127]

**Table 3:** List of top five factors for countries in each World Bank income level identified as having an effect on COVID-19 Vaccine Acceptance. The number of countries in each WB income level for which each factor was identified by previous studies, was recorded, counted, and the percentage countries for that WB income level calculated. No statistical analysis was conducted on this table.

LIC		LMIC		UMIC		HIC	
Factors in ascending order	No. of countries, %	Factors in ascending order	No. of countries, %	Factors in ascending order	No. of countries, %	Factors in ascending order	No. of countries, %
Fear of infection	13, 56.52%	Fear of infection	28, 51.85%	Older persons	32, 65.31%	Fear of infection	25, 42.37%
Older persons	11, 47.83%	Male	22, 40.74%	Male	30, 61.22%	Older persons	23, 38.98%
Male	11, 47.83%	Older	21, 38.89%	Fear of infection	27, 55.10%	Trust	22, 37.29%
High Education	10, 43.48%	High Education	20, 37.04%	High Education	25, 51.02%	Higher income earner	21, 35.59%
Younger	6, 29.09%	Higher income earner	12, 22.22%	Healthcare worker	21, 42.86%	Male	21, 35.59%

**Table 4:** List of top five factors identified that commonly affect VH. The number of countries in each WB income level for which each factor was identified by previous studies, was recorded, counted, and the percentage countries for that WB income level calculated. No statistical analysis was conducted on this table.

LIC		LMIC		UMIC		HIC	
Most frequent factors in descending order	No. of countries, %	Most frequent factors in descending order	No. of countries, %	Most frequent factors in descending order	No. of countries, %	Most frequent factors in descending order	No. of countries, %
Female	17, 73.91%	Female	30, 55.56%	Female	29, 59.18%	Mistrust of vaccine	26, 44.07%
Safety	14, 60.87%	Mistrust of vaccine	29, 53.71%	Mistrust of vaccine	29, 59.18%	Low Education	23, 38.98%
Mistrust of vaccine	14, 60.87%	Younger	21, 38.89%	Low Education	26, 53.06%	Safety	21, 35.59%
Younger	11, 47.83%	Safety	21, 38.89%	Safety	25, 51.02%	Female	19, 32.21%
Low Education	9, 39.13%	Low Education	17, 31.48%	Younger	23, 46.94%	Younger	16, 27.12%

## Data Availability

The data used for this research and the R Scripts used for statistical analysis and generating plots are available upon request from the corresponding author.

## Supplementary Materials

The following supporting information can be downloaded at: https://www.mdpi.com/article/10.3390/vaccines12010034/s1, Table S1: Factors associated with COVID-19 vaccine acceptance that were identified in the selected studies, grouped according to the Health Belief Model, and analyzed in this synthesis. Table S2: Factors associated with COVID-19 vaccine hesitancy that were identified in the selected studies, grouped according to the Health Belief Model, and analyzed in this synthesis.

## References

[1] Filip R., Puscaselu R.G., Anchidin-Norocel L., Dimian M., Savage W.K. (2022). Global Challenges to Public Health Care Systems during the COVID-19 Pandemic: A Review of Pandemic Measures and Problems. J. Pers. Med..

[2] Kawuki J., Chan P.S., Fang Y., Chen S., Mo P.K.H., Wang Z. (2023). Knowledge and Practice of Personal Protective Measures against COVID-19 in Africa: Systematic Review. JMIR Public Health Surveill..

[3] Keleb A., Ademas A., Lingerew M., Sisay T., Berihun G., Adane M. (2021). Prevention Practice of COVID-19 Using Personal Protective Equipment and Hand Hygiene among Healthcare Workers in Public Hospitals of South Wollo Zone, Ethiopia. Front. Public Health.

[4] Lio C.F., Cheong H.H., Lei C.I., Lo I.K., Yao L., Lam C., Leong I.H. (2021). Effectiveness of personal protective health behaviour against COVID-19. BMC Public Health.

[5] Ngwewondo A., Nkengazong L., Ambe L.A., Ebogo J.T., Mba F.M., Goni H.O., Nyunaï N., Ngonde M.C., Oyono J.-L.E. (2020). Knowledge, attitudes, practices of/towards COVID 19 preventive measures and symptoms: A cross-sectional study during the exponential rise of the outbreak in Cameroon. PLoS Negl. Trop. Dis..

[6] Voundi-Voundi E., Songue E., Voundi-Voundi J., Nseme E.G., Abba-Kabir H., Kamgno J. (2023). Factors Associated with COVID-19 Vaccine Hesitancy among Health Personnel in Yaounde, Cameroon. Health Sci. Dis..

[7] Nah S., Williamson L.D., Kahlor L.A., Atkinson L., Ntang-Beb J.-L., Upshaw S.J. (2023). COVID-19 Vaccine Hesitancy in Cameroon: The Role of Medical Mistrust and Social Media Use. J. Health Commun..

[8] Tetsatsi A.C.M., Nguena A.A., Deutou A.L., Talom A.T., Metchum B.T., Tiotsia A.T., Watcho P., Colizzi V. (2023). Factors Associated with COVID-19 Vaccine Refusal: A Community-Based Study in the Menoua Division in Cameroon. Trop. Med. Infect. Dis..

[9] Nuwarda R.F., Ramzan I., Weekes L., Kayser V. (2022). Vaccine Hesitancy: Contemporary Issues and Historical Background. Vaccines.

[10] Lazarus J.V., Diana Romero D., Kopka C.J., Karim S.A., Abu-Raddad L.J., Almeida G., Baptista-Leite R., Barocas J.A., Barreto M.L., Yaneer Bar-Yam Y. (2022). A multinational Delphi consensus to end the COVID-19 public health threat. Nature.

[11] Ayouni I., Maatoug J., Dhouib W., Zammit N., Fredj S.B., Ghammam R., Ghannem H. (2021). Effective public health measures to mitigate the spread of COVID-19: A systematic review. BMC Public Health.

[12] Khairi L.N.H.M., Fahrni M.L., Lazzarino A.I. (2022). The Race for Global Equitable Access to COVID-19 Vaccines. Vaccines.

[13] Doroshenko A. (2021). The Combined Effect of Vaccination and Nonpharmaceutical Public Health Interventions—Ending the COVID-19 Pandemic. JAMA Netw. Open.

[14] Motta M., Sylvester S., Callaghan T., Lunz-Trujillo K. (2021). Encouraging COVID-19 Vaccine Uptake Through Effective Health Communication. Front. Polit. Sci..

[15] Ndwandwe D., Wiysonge C.S. (2021). COVID-19 vaccines. Curr. Opin. Immunol..

[16] Graham B.S. (2020). Rapid COVID-19 vaccine development. Science.

[17] Kashte S., Gulbake A., El-Amin S.F., Gupta A. (2021). COVID-19 vaccines: Rapid development, implications, challenges and future prospects. Human Cell.

[18] Li Y., Tenchov R., Smoot J., Liu C., Watkins S., Zhou Q. (2021). A Comprehensive Review of the Global Efforts on COVID-19 Vaccine Development. ACS Cent. Sci..

[19] VIPER Group COVID19 Vaccine Tracker Team 12 Vaccines Granted Emergency Use Listing (EUL) by WHO. https://covid19.trackvaccines.org/agency/who/.

[20] Quilici S., Smith R., Signorelli C. (2015). Role of vaccination in economic growth. J. Mark. Access Health Policy.

[21] Rodrigues C.M.C., Plotkin S.A. (2020). Impact of Vaccines; Health, Economic and Social Perspectives. Front. Microbiol..

[22] Cadarette D., Ferranna M., Cannon J.W., Abbas K., Giannini F., Zucker L., Bloom D.E. (2023). The full health, economic, and social benefits of prospective Strep A vaccination. NPJ Vaccines.

[23] Decouttere C., De Boeck K., Vandaele N. (2021). Advancing sustainable development goals through immunization: A literature review. Global Health.

[24] Ethgen O., Rémy V., Wargo K. (2018). Vaccination budget in Europe: An update. Hum. Vaccin. Immunother..

[25] Wouters O.J., Shadlen K.C., Salcher-Konrad M., Pollard A.J., Larson H.J., Teerawattananon Y., Jit M. (2021). Challenges in ensuring global access to COVID-19 vaccines: Production, affordability, allocation, and deployment. Lancet.

[26] Duroseau B., Kipshidze N., Limaye R.J. (2023). The impact of delayed access to COVID-19 vaccines in low- and lower-middle-income countries. Front. Public Health.

[27] WHO Vaccine Inequity Undermining Global Economic Recovery. https://www.who.int/news/item/22-07-2021-vaccine-inequity-undermining-global-economic-recovery.

[28] UNICEF COVAX: Ensuring Global Equitable Access to COVID-19 Vaccines. https://www.unicef.org/supply/covax-ensuring-global-equitable-access-covid-19-vaccines.

[29] Nourou M.A. ECOWAS Launches Revolving Fund to Secure 240mln Doses of COVID-19 Vaccine. https://www.ecofinagency.com/public-management/2601-42285-ecowas-launches-revolving-fund-to-secure-240mln-doses-of-covid-19-vaccine.

[30] Boskey E. How the Health Belief Model Influences Your Behaviors. https://www.verywellmind.com/health-belief-model-3132721.

[31] Macdonald N.E., SAGE Working Group on Vaccine Hesitancy (2015). Vaccine hesitancy: Definition, scope and determinants. Vaccine.

[32] Sahakyan S., Gharibyan N., Aslanyan L., Hayrumyan V., Harutyunyan A., Libaridian L., Grigoryan Z. (2023). Multi-Perspective Views and Hesitancy toward COVID-19 Vaccines: A Mixed Method Study. Vaccines.

[33] Kafadar A.H., Tekeli G.G., Jones K.A., Stephan B., Dening T. (2023). Determinants for COVID-19 vaccine hesitancy in the general population: A systematic review of reviews. J. Public Health.

[34] Titanji V.P.K., Ghogomu S.M., Dinga J.N., Nzwendji J.G., Mbah D.A. (2022). Scientific evidence-based response to pandemics in resource-limited countries with particular reference to the COVID-19: The case of Cameroon. J. Cameroon Acad. Sci..

[35] Dinga J.N., Titanji V.P.K. (2022). The challenge of Vaccine hesitancy and rejection, with a focus on Cameroon and Africa: A mini review. J. Cameroon Acad. Sci..

[36] Kabakama S., Konje E.T., Dinga J.N., Kishamawe C., Morhason-Bello I., Hayombe P., Adeyemi O., Chimuka E., Lumu I., Amuasi J. (2022). Commentary on COVID-19 Vaccine Hesitancy in sub-Saharan Africa. Trop. Med. Infect. Dis..

[37] İkiışık H., Sezerol M.A., Taşçı Y., Maral I. (2022). COVID-19 vaccine hesitancy and related factors among primary healthcare workers in a district of Istanbul: A cross-sectional study from Turkey. Fam. Med. Community Health.

[38] Rode O.Đ., Bodulić K., Zember S., Balent N.C., Novokmet A., Čulo M., Rašić Ž., Mikulić R., Markotić A. (2022). Decline of Anti-SARS-CoV-2 IgG Antibody Levels 6 Months after Complete BNT162b2 Vaccination in Healthcare Workers to Levels Observed Following the First Vaccine Dose. Vaccines.

[39] Jeyanathan M., Afkhami S., Smaill F., Miller M.S., Lichty B.D., Xing Z. (2020). Immunological considerations for COVID-19 vaccine strategies. Nat. Rev. Immunol..

[40] Zimmermann P., Curtis N. (2019). Factors That Influence the Immune Response to Vaccination. Clin. Microbiol. Rev..

[41] Hertz T., Levy S., Ostrovsky D., Oppenheimer H., Zismanov S., Kuzmina A., Friedman L.M., Trifkovic S., Brice D., Chun-Yang L.C. (2023). Correlates of protection for booster doses of the SARS-CoV-2 vaccine BNT162b2. Nat. Commun..

[42] Eguavoen A., Larson H., Chinye-Nwoko F., Ojeniyi T. (2023). Reducing COVID-19 vaccine hesitancy and improving vaccine uptake in Nigeria. J. Public Health Afr..

[43] National Academies of Sciences, Engineering, and Medicine, Health and Medicine Division, Board on Population Health and Public Health Practice, Board on Health Sciences Policy, Committee on Equitable Allocation of Vaccine for the Novel Coronavirus (2020). Achieving Acceptance of COVID-19 Vaccine. Framework for Equitable Allocation of COVID-19 Vaccine.

[44] Cascini F., Pantovic A., Al-Ajlouni Y., Failla G., Ricciardi W. (2021). Attitudes, acceptance and hesitancy among the general population worldwide to receive the COVID-19 vaccines and their contributing factors: A systematic review. EClinicalMedicine.

[45] Wiysonge C.S., Ndwandwe D., Ryan J., Jaca A., Batouré O., Anya B.-P.M., Cooper S. (2022). Vaccine hesitancy in the era of COVID-19: Could lessons from the past help in divining the future?. Hum. Vaccines Immunother..

[46] Gerretsen P., Kim J., Quilty L., Wells S., Brown E.E., Agic B., Pollock B.G., Graff-Guerrero A. (2021). Vaccine Hesitancy Is a Barrier to Achieving Equitable Herd Immunity among Racial Minorities. Front. Med..

[47] Hess S., Lancsar E., Mariel P., Meyerhoff J., Song F., Broek-Altenburg E.V.D., Alaba O.A., Amaris G., Arellana J., Basso L.J. (2022). The path towards herd immunity: Predicting COVID-19 vaccination uptake through results from a stated choice study across six continents. Soc. Sci. Med..

[48] Borga L.G., Clark A.E., D’Ambrosio C., Lepinteur A. (2022). Characteristics associated with COVID-19 vaccine hesitancy. Sci. Rep..

[49] Schwarzinger M., Watson V., Arwidson P., Alla F., Luchini S. (2021). COVID-19 vaccine hesitancy in a representative working-age population in France: A survey experiment based on vaccine characteristics. Lancet Public Health.

[50] Hassan W., Kazmi S.K., Tahir M.J., Ullah I., Royan H.A., Fahriani M., Nainu F., Rosa S.G. (2021). Global acceptance and hesitancy of COVID-19 vaccination: A narrative review. Narra J..

[51] Galagali P.M., Kinikar A.A., Kumar V.S. (2022). Vaccine Hesitancy: Obstacles and Challenges. Curr. Pediatr. Rep..

[52] Sallam M. (2021). COVID-19 Vaccine Hesitancy Worldwide: A Concise Systematic Review of Vaccine Acceptance Rates. Vaccines.

[53] Lohiniva A.-L., Pensola A., Hyökki S., Sivelä J., Härmä V., Tammi T. (2023). Identifying factors influencing COVID-19 vaccine uptake in Finland—A qualitative study using social media data. Front. Public Health.

[54] Sharma E., Mondal S., Das S., Vrana V.G. (2023). Scale Development for COVID-19 Vaccine Hesitancy by Integration of Socio-Demographic and Psychological Factors. Vaccines.

[55] Nossier S.A. (2021). Vaccine hesitancy: The greatest threat to COVID-19 vaccination programs. J. Egypt. Public Health. Assoc..

[56] Lee S.K., Sun J., Jang S., Connelly S. (2022). Misinformation of COVID-19 vaccines and vaccine hesitancy. Sci. Rep..

[57] Yoshida M., Kobashi Y., Kawamura T., Shimazu Y., Nishikawa Y., Omata F., Zhao T., Yamamoto C., Kaneko Y., Nakayama A. (2022). Factors Associated with COVID-19 Vaccine Booster Hesitancy: A Retrospective Cohort Study, Fukushima Vaccination Community Survey. Vaccines.

[58] Raut A., Samad A., Verma J., Kshirsagar P. (2023). Acceptance, hesitancy and refusal towards COVID-19 vaccination. Clin. Epidemiol. Glob. Health.

[59] Hayawi K., Shahriar S., Serhani M.A., Alashwal H., Masud M.M. (2021). Vaccine versus Variants (3Vs): Are the COVID-19 Vaccines Effective against the Variants? A Systematic Review. Vaccines.

[60] Malik J.A., Ahmed S., Mir A., Shinde M., Bender O., Alshammari F., Ansari M., Anwar S. (2022). The SARS-CoV-2 mutations versus vaccine effectiveness: New opportunities to new challenges. J. Infect. Public Health.

[61] Young M., Crook H., Scott J., Edison P. (2022). COVID-19: Virology, variants, and vaccines. BMJ Med..

[62] Willett B.J., Grove J., MacLean O.A., Wilkie C., De Lorenzo G., Furnon W., Cantoni D., Scott S., Logan N., Ashraf S. (2022). SARS-CoV-2 Omicron is an immune escape variant with an altered cell entry pathway. Nat. Microbiol..

[63] Moher D., Liberati A., Tetzlaff J., Altman D.G., The PRISMA Group (2009). Preferred reporting items for systematic reviews and meta-analyses: The PRISMA statement. BMJ.

[64] van Rompaey C., Metreau E., Hamadeh World Bank Group Country Classifications by Income Level for FY24 (1 July 2023–30 June 2024). https://blogs.worldbank.org/opendata/new-world-bank-group-country-classifications-income-level-fy24.

[65] The World Bank Group World Bank Open Data: Current Health Expenditure (% of GDP). World Bank Open Data..

[66] World Health Organization Immunization Expenditure Indicators. https://www.who.int/teams/immunization-vaccines-and-biologicals/vaccine-access/planning-and-financing/immunization-financing-indicators.

[67] Mckellar K., Sillence E., Mckellar K., Sillence E. (2020). Current Research on Sexual Health and Teenagers: Health Belief Model. Teenagers, Sexual Health Information and the Digital Age.

[68] Downes M.J., Brennan M.L., Williams H.C., Dean R.S. (2016). Development of a critical appraisal tool to assess the quality of cross-sectional studies (AXIS). BMJ Open.

[69] Azimi M., Yadgari M.Y., Atiq M.A. (2023). Acceptance and Hesitancy Toward the COVID-19 Vaccine among Medical Students in Kabul, Afghanistan. Infect. Drug Resist..

[70] Abay E.S., Belew M.D., Ketsela B.S., Mengistu E.E., Getachew L.S., Teferi Y.A., Zerihun A.B. (2022). Assessment of attitude towards COVID-19 vaccine and associated factors among clinical practitioners in Ethiopia: A cross-sectional study. PLoS ONE.

[71] Ackah B.B.B., Woo M., Stallwood L., Fazal Z.A., Okpani A., Ukah U.V., Adu P.A. (2022). COVID-19 vaccine hesitancy in Africa: A scoping review. Glob. Health Res. Policy.

[72] Africa CDC (2021). COVID-19 Vaccine Perceptions: A 15-Country Study. CDC Africa COVID-19 Vaccine Perceptions..

[73] Ahmed M.A.M., Colebunders R., Gele A.A., Farah A.A., Osman S., Guled I.A., Abdullahi A.A.M., Hussein A.M., Ali A.M., Fodjo J.N.S. (2021). COVID-19 Vaccine Acceptability and Adherence to Preventive Measures in Somalia: Results of an Online Survey. Vaccines.

[74] Eberwein J.D., Edochie I., Newhouse D., Cojocaru A., Deudibe G., Kakietek J., Kim Y.S., Montes J. (2022). COVID-19 Vaccine Hesitancy in 53 Developing Countries: Levels, Trends, and Reasons for Hesitancy.

[75] De Figueiredo A., Simas C., Larson H.J. (2023). COVID-19 vaccine acceptance and its socio-demographic and emotional determinants: A multi-country cross-sectional study. Vaccine.

[76] De Sousa Á.F.L., Jules Ramon Brito Teixeira J.R.B., Lua I., Souza F.D.O., Ferreira A.J.F., Schneider G., De Carvalho H.E.F., De Oliveira L.B., Lima S.V.M.A., De Sousa A.R. (2021). Determinants of COVID-19 Vaccine Hesitancy in Portuguese-Speaking Countries: A Structural Equations Modeling Approach. Vaccines.

[77] Dereje N., Tesfaye A., Tamene B., Alemeshet D., Abe H., Tesfa N., Gideon S., Biruk T., Lakew Y. (2022). COVID-19 vaccine hesitancy in Addis Ababa, Ethiopia: A mixed-method study. BMJ Open.

[78] Ditekemena J.D., Nkamba D.M., Mutwadi A., Mavoko H.M., Fodjo J.N.S., Luhata C., Obimpeh M., Hees S.V., Nachega J.B., Colebunders R. (2021). COVID-19 Vaccine Acceptance in the Democratic Republic of Congo: A Cross-Sectional Survey. Vaccines.

[79] Echoru I., Ajambo P.D., Keirania E., Bukenya E.E.M. (2021). Sociodemographic factors associated with acceptance of COVID-19 vaccine and clinical trials in Uganda: A cross-sectional study in western Uganda. BMC Public Health.

[80] Kanyanda S., Markhof Y., Wollburg P., Zezza A. (2021). Acceptance of COVID-19 vaccines in sub-Saharan Africa: Evidence from six national phone surveys. BMJ Open.

[81] Mebarki B., Argoub M., Mokdad M., Mebarki I., Merah A. (2023). COVID-19 Vaccine Hesitancy Behaviour among Algerian Adults at the Onset of the Fourth Wave of Corona Virus Pandemic. ResearchSquare.

[82] Mesele M. (2021). COVID-19 Vaccination Acceptance and Its Associated Factors in Sodo Town, Wolaita Zone, Southern Ethiopia: Cross-Sectional Study. Infect. Drug Resist..

[83] Mohammed R., Nguse T.M., Habte B.M., Fentie A.M., Gebretekle G.B. (2021). COVID-19 vaccine hesitancy among Ethiopian healthcare workers. PLoS ONE.

[84] Mose A., Yeshaneh A. (2021). COVID-19 Vaccine Acceptance and Its Associated Factors among Pregnant Women Attending Antenatal Care Clinic in Southwest Ethiopia: Institutional-Based Cross-Sectional Study. Int. J. Gen. Med..

[85] Patwary M.M., Bardhan M., Haque Z., Sultana R., Alam M.A., Browning M.H.E.M. (2022). COVID-19 Vaccine Acceptance Rate and Its Factors among Healthcare Students: A Systematic Review with Meta-Analysis. Vaccines.

[86] Qunaibi E.A., Helmy M., Basheti I., Sultan I. (2021). A high rate of COVID-19 vaccine hesitancy in a large-scale survey on Arabs. eLife.

[87] Riad A., Abdulqader H., Morgado M., Domnori S., Koščík M., Mendes J., Klugar M., Kateeb E., IADS-SCORE (2021). Global Prevalence and Drivers of Dental Students’ COVID-19 Vaccine Hesitancy. Vaccines.

[88] Rice D.R., Balamo A., Thierry A.-R., Gueral A., Fidele D., Mateen F.J., Sakadi F., Francis J.M. (2022). COVID-19 vaccine acceptance and hesitancy in N’Djamena, Chad: A cross-sectional study of patients, community members, and healthcare workers. PLoS Glob. Public Health.

[89] Sallam M., Al-Sanafi M., Sallam M. (2022). A Global Map of COVID-19 Vaccine Acceptance Rates per Country: An Updated Concise Narrative Review. J. Multidiscip. Healthc..

[90] Arce J.S.S., Warren S.S., Meriggi N.F., Scacco A., McMurry N., Voors M., Syunyaev G., Malik A.A., Aboutajdine S., Adeojo O. (2021). COVID-19 vaccine acceptance and hesitancy in low- and middle-income countries. Nat. Med..

[91] Dzomo G.R.T., Mbario E., Djarma O., Soumbatingar N., Madengar M., Djimera N., Djindimadje A., Nguemadjita C., Nassaringar G., Bernales M. (2023). Predictors of COVID-19 vaccine hesitancy in Chad: A cross-sectional study. Front. Public Health.

[92] Ajonina-Ekoti I.U., Ware K.B., Nfor C.K., Akomoneh E.A., Djam A., Chia-Garba M., Wepnyu G.N., Awambeng D., Abendong K., Manjong F.T. (2022). COVID-19 perceptions and vaccine hesitancy: Acceptance, attitude, and barriers among Cameroonians. J. Am. Pharm. Assoc..

[93] Ali M., Hossain A. (2021). What is the extent of COVID-19 vaccine hesitancy in Bangladesh? A cross-sectional rapid national survey. BMJ Open.

[94] Amour M.A., Mboya I.B., Ndumwa H.P., Kengia J.T., Metta E., Njiro B.J., Nyamuryekung’e K.K., Mhamilawa L.E., Shayo E.H., Ngalesoni F. (2023). Determinants of COVID-19 Vaccine Uptake and Hesitancy among Healthcare Workers in Tanzania: A Mixed-Methods Study. COVID.

[95] Avahoundje E.M., Dossou J.-P., Vigan A., Gaye I., Agossou C., Boyi C., Bello K., Mikponhoue J., Ba M.F., Faye A. (2022). Factors associated with COVID-19 vaccine intention in Benin in 2021: A cross-sectional study. Vaccine X.

[96] Ba M.F., Faye A., Kane B., Diallo A.I., Junot A., Gaye I., Bonnet E., Ridde V. (2022). Factors associated with COVID-19 vaccine hesitancy in Senegal: A mixed study. Hum. Vaccines Immunother..

[97] Dinga J.N., Sinda L.K., Titanji V.P.K. (2021). Assessment of Vaccine Hesitancy to a COVID-19 Vaccine in Cameroonian Adults and Its Global Implication. Vaccines.

[98] Dinga J.N., Njoh A.A., Gamua S.D., Muki S.E., Titanji V.P.K. (2022). Factors Driving COVID-19 Vaccine Hesitancy in Cameroon and Their Implications for Africa: A Comparison of Two Cross-Sectional Studies Conducted 19 Months Apart in 2020 and 2022. Vaccines.

[99] Hossain M.B., Alam M.Z., Islam M.D., Sultan S., Faysal M.M., Rima S., Hossain M.A., Mamun A. (2021). COVID-19 vaccine hesitancy among the adult population in Bangladesh: A nationwide cross-sectional survey. PLoS ONE.

[100] Hossain S., Islam M.S., Pardhan S., Banik R., Ahmed A., Islam M.Z., Mahabub M.S., Sikder M.T. (2022). Beliefs, barriers and hesitancy towards the COVID-19 vaccine among Bangladeshi residents: Findings from a cross-sectional study. PLoS ONE.

[101] Kacimi S.E.O., Klouche-Djedid S.N., Riffi O., Belaouni H.A., Yasmin F., Essar M.Y., Taouza F.A., Belakhdar Y., Fellah S.C., Benmelouka A.Y. (2022). Determinants of COVID-19 Vaccine Engagement in Algeria: A Population-Based Study With Systematic Review of Studies From Arab Countries of the MENA Region. Front. Public Health.

[102] Lamptey E., Serwaa D., Appiah A.B. (2021). A nationwide survey of the potential acceptance and determinants of COVID-19 vaccines in Ghana. Clin. Exp. Vaccine Res..

[103] Lazarus J.V., Wyka K., White T.M., Picchio C.A., Gostin L.O., Larson H.J., Rabin K., Ratzan S.C., Kamarulzaman A., El-Mohandes A. (2023). A survey of COVID-19 vaccine acceptance across 23 countries in 2022. Nat. Med..

[104] Lounis M., Abdelhadi S., Rais M.A., Bencherit D., Sallam M. (2022). Intention to get COVID-19 vaccination and its associated predictors: A cross-sectional study among the general public in Algeria. Vacunas.

[105] Marzo R.R., Sami W., Alam M.Z., Acharya S., Jermsittiparsert K., Songwathana K., Pham N.T., Respati T., Faller E.M., Baldonado A.M. (2022). Hesitancy in COVID-19 vaccine uptake and its associated factors among the general adult population: A cross-sectional study in six Southeast Asian countries. Trop. Med. Health.

[106] Mudenda S., Hikaambo C.N., Daka V., Chileshe M., Mfune R.L., Kampamba M., Kasanga M., Phiri M., Mufwambi W., Banda M. (2022). Prevalence and factors associated with COVID-19 vaccine acceptance in Zambia: A web-based cross-sectional study. Pan Afr. Med. J..

[107] Padonou S., Glèlè C.K., Accrombessi M., Adegbite B., Dangbenon E., Bah H., Akogbeto E., Chabi A.B., Kaucley L., Sourakatou S. (2023). Assessment of COVID-19 Vaccine Acceptance and Its Associated Factors during the Crisis: A Community-Based Cross-Sectional Study in Benin. Vaccines.

[108] Puertas E.B., Velandia-Gonzalez M., Vulanovic L., Bayley L., Broome K., Ortiz C., Rise N., Antelo M.V., Rhoda D.A. (2022). Concerns, attitudes, and intended practices of Caribbean healthcare workers concerning COVID-19 vaccination: A cross-sectional study. Lancet Reg. Health-Am..

[109] Daşıkan Z., Ekrem E.C., Kıratlı D. (2023). COVID-19 Vaccine Acceptance among Pregnant, Lactating, and Nonpregnant Women of Reproductive Age in Turkey: A Cross-Sectional Analytical Study. Disaster Med. Public Health Prep..

[110] Doran J., Seyidov N., Mehdiyev S., Gon G., Kissling E., Herdman T., Suleymanova J., Couthino Rehse A.P.C., Pebody R., Katz M.A. (2022). Factors associated with early uptake of COVID-19 vaccination among healthcare workers in Azerbaijan, 2021. Influenza Respir. Viruses.

[111] Gentile A., Pacchiotti A.C., Giglio N., Nolte M.F., Talamona N., Rogers V., Berenstein A., Castellano V.E. (2021). Vaccine hesitancy in Argentina: Validation of WHO scale for parents. Vaccine.

[112] Jorgensen P., Schmid A., Sulo J., Preza I., Hasibra I., Kissling E., Fico A., Sridhar S., Rubin-Smith J.E., Kota M. (2023). Factors associated with receipt of COVID-19 vaccination and SARS-CoV-2 seropositivity among healthcare workers in Albania (February 2021–June 2022): Secondary analysis of a prospective cohort study. Lancet Reg. Health-Eur..

[113] Miljanovic S.M., Cvjetkovic S., Jeremic-Stojkovic V., Mandic-Rajcevic S. (2022). COVID-19 vaccine hesitancy in five Western Balkan countries. Eur. J. Public Health.

[114] Šljivo A., Ćetković A., Abdulkhaliq A. (2021). COVID-19 vaccination knowledge, attitudes and practices among residents of Bosnia and Herzegovina during the third wave of COVID-19 outbreak. Ann. Ig. Med. Prev. Comunità.

[115] Cuschieri S., Grech V. (2022). A comparative assessment of attitudes and hesitancy for influenza vis-à-vis COVID-19 vaccination among healthcare students and professionals in Malta. J. Public Health.

[116] Di Valerio Z., Montalti M., Guaraldi F., Tedesco D., Nreu B., Mannucci E., Monami M., Gori D. (2022). Trust of Italian healthcare professionals in COVID-19 (anti-SARS-CoV-2) vaccination. Ann. Ig..

[117] Gagneux-Brunon A., Detoc M., Bruel S., Tardy B., Rozaire O., Frappe P., Botelho-Nevers E. (2021). Intention to get vaccinations against COVID-19 in French healthcare workers during the first pandemic wave: A cross-sectional survey. J. Hosp. Infect..

[118] Galanis P., Moisoglou I., Vraka I., Siskou O., Konstantakopoulou O., Katsiroumpa A., Kaitelidou D. (2022). Predictors of COVID-19 Vaccine Uptake in Healthcare Workers: A Cross-Sectional Study in Greece. J. Occup. Environ. Med..

[119] Kelly B.J., Southwell B.G., McCormack L.A., Bann C.M., MacDonald P.D.M., Frasier A.M., Bevc C.A., Brewer N.T., Squiers L.B. (2021). Predictors of willingness to get a COVID-19 vaccine in the U.S. BMC Infect. Dis..

[120] King I., Heidler P., Marzo R.R. (2021). The Long and Winding Road: Uptake, Acceptability, and Potential Influencing Factors of COVID-19 Vaccination in Austria. Vaccines.

[121] Lindholt M.F., Jørgensen F., Bor A., Petersen M.B. (2021). Public acceptance of COVID-19 vaccines: Cross-national evidence on levels and individual-level predictors using observational data. BMJ Open.

[122] Murphy J., Vallières F., Bentall R.P., Shevlin M., McBride O., Hartman T.K., McKay R., Bennett K., Mason L., Gibson-Miller J. (2021). Psychological characteristics associated with COVID-19 vaccine hesitancy and resistance in Ireland and the United Kingdom. Nat. Commun..

[123] Patwary M.M., Alam M.A., Bardhan M., Disha A.S., Haque M.Z., Billah S.M., Kabir M.P., Browning M.H.E.M., Rahman M.M., Parsa A. (2022). COVID-19 Vaccine Acceptance among Low- and Lower-Middle-Income Countries: A Rapid Systematic Review and Meta-Analysis. Vaccines.

[124] Robertson E., Reeve K.S., Niedzwiedz C.L., Moore J., Blake M., Green M., Katikireddi S.V., Benzeval M.J. (2021). Predictors of COVID-19 vaccine hesitancy in the UK household longitudinal study. Brain Behav. Immun..

[125] Stamm T.A., Partheymüller J., Mosor E., Ritschl V., Kritzinger S., Eberl J.-M. (2022). Coronavirus vaccine hesitancy among unvaccinated Austrians: Assessing underlying motivations and the effectiveness of interventions based on a cross-sectional survey with two embedded conjoint experiments. Lancet Reg. Health Eur..

[126] UNICEF (2022). COVID-19 Vaccine Hesitancy Survey Report 2022—Antigua and Barbuda. https://www.unicef.org/easterncaribbean/reports/covid-19-vaccine-hesitancy-survey-report-2022-antigua-and-barbuda.

[127] Verger P., Scronias D., Dauby N., Adedzi K.A., Gobert C., Bergeat M., Gagneur A., Dubé E. (2021). Attitudes of healthcare workers towards COVID-19 vaccination: A survey in France and French-speaking parts of Belgium and Canada, 2020. Eurosurveillance.

[128] South A. (2011). Rworldmap: A new R package for mapping global data. R J..

[129] Baghani M., Fathalizade F., Loghman A.H., Samieefar N., Ghobadinezhad F., Rashedi R., Baghsheikhi H., Sodeifian F., Rahimzadegan M., Akhlaghdoust M. (2023). COVID-19 vaccine hesitancy worldwide and its associated factors: A systematic review and meta-analysis. Sci. One Health.

[130] Mengistu D.A., Demmu Y.M., Asefa Y.A. (2022). Global COVID-19 vaccine acceptance rate: Systematic review and meta-analysis. Front. Public Health.

[131] Wang Q., Simeng Hu S., Du F., Zang S., Xing Y., Qu Z., Zhang X., Lin L., Hou Z. (2022). Mapping global acceptance and uptake of COVID-19 vaccination: A systematic review and meta-analysis. Commun. Med..

[132] Noushad M., Rastam S., Nassani M.Z., Al-Saqqaf I.S., Hussain M., Yaroko A.A., Arshad M.A., Kirfi A.M., Koppolu P., Niazi F.H. (2022). A Global Survey of COVID-19 Vaccine Acceptance among Healthcare Workers. Front. Public Health.

[133] Terry E., Cartledge S., Damery S., Greenfield S. (2022). Factors associated with COVID-19 vaccine intentions during the COVID-19 pandemic; a systematic review and meta-analysis of cross-sectional studies. BMC Public Health.

[134] Lazarus J.V., Wyka K., White T.M., Picchio C.A., Rabin K., Ratzan S.C., Leigh J.P., Hu J., El-Mohandes A. (2022). Revisiting COVID-19 vaccine hesitancy around the world using data from 23 countries in 2021. Nat. Commun..

[135] Bono S.A., Villela E.F.M., Siau C.S., Chen W.S., Pengpid S., Hasan M.T., Sessou P., Ditekemena J.D., Amodan B.O., Hosseinipour M.C. (2021). Factors Affecting COVID-19 Vaccine Acceptance: An International Survey among Low- and Middle-Income Countries. Vaccines.

[136] Mekonnen B.D., Mengistu B.A. (2022). COVID-19 vaccine acceptance and its associated factors in Ethiopia: A systematic review and meta-analysis. Clin. Epidemiol. Glob. Health.

[137] Norhayati M.N., Yusof R.C., Azman Y.M. (2022). Systematic Review and Meta-Analysis of COVID-19 Vaccination Acceptance. Front. Med..

[138] Kaadan M.I., Abdulkarim J., Chaar M., Zayegh O., Keblawi M.A. (2021). Determinants of COVID-19 vaccine acceptance in the Arab world: A cross-sectional study. Glob. Health Res. Policy.

[139] Roy D.N., Biswas M., Islam E., Azam S. (2022). Potential factors influencing COVID-19 vaccine acceptance and hesitancy: A systematic review. PLoS ONE.

[140] Shakeel C.S., Mujeeb A.A., Mirza M.S., Chaudhry B., Khan S.J. (2022). Global COVID-19 Vaccine Acceptance: A Systematic Review of Associated Social and Behavioral Factors. Vaccines.

[141] Fattah A., Mohammadtaghizadeh M., Azadi H. (2022). Factors Associated with COVID-19 Vaccine Acceptance Worldwide: A Rapid Review. Med. Educ. Bull..

[142] Ibrahim F.M., Fadila D.E., Elmawla D.A.E.A. (2023). Older adults’ acceptance of the COVID-19 vaccine: Application of the health belief model. Nurs. Open.

[143] Bullivant B., Bolsewicz K.T., King C., Steffens M.S. (2023). COVID-19 vaccination acceptance among older adults: A qualitative study in New South Wales, Australia. Public Health Pract..

[144] Sezerol M.A., Davun S. (2023). COVID-19 Vaccine Booster Dose Acceptance among Older Adults. Vaccines.

[145] Zhang Y., Wang Y., Ning G., He P., Wang W. (2022). Protecting older people: A high priority during the COVID-19 pandemic. Lancet.

[146] Malani P.N., Solway E., Kullgren J.T. (2020). Older Adults’ Perspectives on a COVID-19 Vaccine. JAMA Health Forum.

[147] Weyand C.M., Goronzy J.J. (2016). Aging of the Immune System. Mechanisms and Therapeutic Targets. Ann. Am. Thorac. Soc..

[148] Li Y., Wang C., Peng M. (2021). Aging Immune System and Its Correlation With Liability to Severe Lung Complications. Front. Public Health.

[149] Grifoni A., Alonzi T., Alter G., Noonan D.M., Landay A.L., Albini A., Goletti D. (2023). Impact of aging on immunity in the context of COVID-19, HIV, and tuberculosis. Front. Immunol..

[150] Bajaj V., Gadi N., Spihlman A.P., Wu S.C., Choi C.H., Moulton V.R. (2021). Aging, Immunity, and COVID-19: How Age Influences the Host Immune Response to Coronavirus Infections?. Front. Physiol..

[151] Kwetkat A., Heppner H.J., Weinberger B. (2020). Comorbidities in the Elderly and Their Possible Influence on Vaccine Response. Interdisciplinary Topics in Gerontology and Geriatrics.

[152] Bartleson J.M., Radenkovic D., Covarrubias A.J., Furman D., Winer D.A., Verdin E. (2021). SARS-CoV-2, COVID-19 and the aging immune system. Nat. Aging.

[153] Mueller A.L., McNamara M.S., Sinclair D.A. (2020). Why does COVID-19 disproportionately affect older people?. Aging.

[154] Jergović M., Coplen C.P., Uhrlaub J.L., Nikolich-Žugich J. (2021). Immune response to COVID-19 in older adults. J. Heart Lung Transplant..

[155] Colebunders R., Fodjo J.N.S. (2022). COVID-19 in Low and Middle Income Countries. Pathogens.

[156] Cénat J.M., Noorishad P.-G., Farahi S.M.M.M., Darius W.P., Aouame A.M.E., Onesi O., Broussard C., Furyk S.E., Yaya S., Caulley L. (2023). Prevalence and factors related to COVID-19 vaccine hesitancy and unwillingness in Canada: A systematic review and meta-analysis. J. Med. Virol..

[157] Kigongo E., Kabunga A., Tumwesigye R., Musinguzi M., Izaruku R., Acup W. (2023). Prevalence and predictors of COVID-19 vaccination hesitancy among healthcare workers in Sub-Saharan Africa: A systematic review and meta-analysis. PLoS ONE.

[158] Dey S., Kusuma Y.S., Kant S., Kumar D., Gopalan R.B., Sridevi P., Aggarwal S. (2023). COVID-19 vaccine acceptance and hesitancy in Indian context: A systematic review and meta-analysis. Pathog. Glob. Health.

[159] Pekcan B., Cai P., Olivas P. (2022). COVID-19 Vaccine Hesitancy and Acceptance in the Global Context: A Systematic Review and Meta-Analysis. Columbia Univ. J. Glob. Heal..

[160] Islam M.M., Yunus M.Y., Akib M.S., Iqbal M.R., Khan M. (2023). Prevalence of COVID-19 Vaccine Hesitancy in South Asia: A Systematic Review and Meta-Analysis. J. Popul. Soc. Stud..

[161] Gulle B.T., Oren M.M., Dal T. (2023). COVID-19 vaccine hesitancy in Turkey: A systematic review and meta-analysis. Epidemiol. Infect..

[162] Page M., Higgins J., Sterne J. (2023). Chapter 13: Assessing risk of bias due to missing results in a synthesis. Cochrane Handbook for Systematic Reviews of Interventions Version 6.4 (Updated August 2023).

[163] Nichol B., McCready J.L., Steen M., Unsworth J., Simonetti V., Tomietto M. (2023). Barriers and facilitators of vaccine hesitancy for COVID-19, influenza, and pertussis during pregnancy and in mothers of infants under two years: An umbrella review. PLoS ONE.

[164] Tostrud L., Thelen J., Palatnik A. (2022). Models of determinants of COVID-19 vaccine hesitancy in non-pregnant and pregnant population: Review of current literature. Hum. Vaccines Immunother..

[165] Lalot F., Abrams D., Heering M.S., Babaian J., Ozkececi H., Peitz L., Hayon K.D., Broadwood J. (2023). Distrustful Complacency and the COVID -19 Vaccine: How Concern and Political Trust Interact to Affect Vaccine Hesitancy. Political Psychol..

[166] Adeyanju G.C., Adeyanju G.C., Engel E., Koch L., Ranzinger T., Shahid I.B.M., Head M.G., Eitze S., Betsch C. (2021). Determinants of influenza vaccine hesitancy among pregnant women in Europe: A systematic review. Eur. J. Med. Res..

[167] Gary J. (2006). The Essential Impact of Context on Organizational Behavior. Acad. Manag. Rev..

[168] Bajos N., Spire A., Silberzan L., Sireyjol A., Jusot F., Meyer L., Franck J.-E., Warszawski J., The EpiCov Study Group (2022). When Lack of Trust in the Government and in Scientists Reinforces Social Inequalities in Vaccination Against COVID-19. Front. Public Health.

[169] Wynen J., De Beeck S.O., Verhoest K., Glavina M., Six F., Van Damme P., Beutels P., Hendrickx G., Pepermans K. (2022). Taking a COVID-19 Vaccine or Not? Do Trust in Government and Trust in Experts Help Us to Understand Vaccination Intention?. Adm. Soc..

[170] Choi Y., Fox A.M. (2022). Mistrust in public health institutions is a stronger predictor of vaccine hesitancy and uptake than Trust in Trump. Soc. Sci. Med..

[171] Jennings W., Valgarðsson V., McKay L., Stoker G., Mello E., Baniamin H.M. (2023). Trust and vaccine hesitancy during the COVID-19 pandemic: A cross-national analysis. Vaccine X.

[172] Carrieri V., Guthmuller S., Wübker A. (2023). Trust and COVID-19 vaccine hesitancy. Sci. Rep..

[173] Harris O.O., Perry T.E., Johnson J.K., Lichtenberg P., Washington T., Kitt B., Shaw M., Keiser S., Tran T., Vest L. (2023). Understanding the concept of trust and other factors related to COVID-19 vaccine intentions among Black/African American older adults prior to vaccine development. SSM-Qual. Res. Health.

[174] Silver D., Kim Y., McNeill E., Piltch-Loeb R., Wang V., Abramson D. (2022). Association between COVID-19 vaccine hesitancy and trust in the medical profession and public health officials. Prev. Med..

[175] Yamanis T., Carlitz R., Gonyea O., Skaff S., Kisanga N., Mollel H. (2023). Confronting ‘chaos’: A qualitative study assessing public health officials’ perceptions of the factors affecting Tanzania’s COVID-19 vaccine rollout. BMJ Open.

[176] Bolsen T., Palm R. (2022). Politicization and COVID-19 vaccine resistance in the U.S. Progress in Molecular Biology and Translational Science.

[177] Backhaus I., Hoven H., Kawachi I. (2023). Far-right political ideology and COVID-19 vaccine hesitancy: Multilevel analysis of 21 European countries. Soc. Sci. Med..

[178] Albrecht D. (2022). Vaccination, politics and COVID-19 impacts. BMC Public Health.

[179] Cata-Preta B.D.O., Wehrmeister F.C., Santos T.M., Barros A.J.D., Victora C.G. (2021). Patterns in Wealth-related Inequalities in 86 Low- and Middle-Income Countries: Global Evidence on the Emergence of Vaccine Hesitancy. Am. J. Prev. Med..

[180] Anderson I. Rich-Country Health Care Systems: Global Lessons. DEVPOLICYBLOG. https://devpolicy.org/rich-country-health-care-systems-global-lessons-20210831/.

[181] Peano A., Politano G., Gianino M.M. (2023). Determinants of COVID-19 vaccination worldwide: WORLDCOV, a retrospective observational study. Front. Public Health.

[182] Moradpour J., Shajarizadeh A., Carter J., Chit A., Grootendorst P. (2023). The impact of national income and vaccine hesitancy on country-level COVID-19 vaccine uptake. PLoS ONE.

[183] Basak P., Abir T., Al Mamun A., Zainol N.R., Khanam M., Haque M.R., Milton A.H., Agho K.E. (2022). A Global Study on the Correlates of Gross Domestic Product (GDP) and COVID-19 Vaccine Distribution. Vaccines.

[184] Wang H., Yu B., Chen X., Yan H. (2023). Global pattern and determinants of coronavirus disease 2019 (COVID-19) vaccine coverage and progression: A global ecological study. Glob. Health J..

[185] Allen J.D., Fu Q., Shrestha S., Nguyen K.H., Stopka T.J., Cuevas A., Corlin L. (2022). Medical mistrust, discrimination, and COVID-19 vaccine behaviors among a national sample U.S. adults. SSM-Popul. Health.

[186] Charura D., Hill A.P., Etherson M.E. (2023). COVID-19 Vaccine Hesitancy, Medical Mistrust, and Mattering in Ethnically Diverse Communities. J. Racial Ethn. Health Disparities.

[187] Lalumera E. (2018). Trust in health care and vaccine hesitancy. Estetica.

[188] Leask J., Carlson S.J., Attwell K., Clark K.K., Kaufman J., Hughes C., Frawley J., Cashman P., Seal H., Wiley K. (2021). Communicating with patients and the public about COVID-19 vaccine safety: Recommendations from the Collaboration on Social Science and Immunisation. Med. J. Aust..

[189] Kaufman J., Tuckerman J., Danchin M. (2022). Overcoming COVID-19 vaccine hesitancy: Can Australia reach the last 20 percent?. Expert Rev. Vaccines.

[190] Qin C., Deng J., Du M., Liu Q., Wang Y., Yan W., Liu M., Liu J. (2023). Pandemic Fatigue and Vaccine Hesitancy among People Who Have Recovered from COVID-19 Infection in the Post-Pandemic Era: Cross-Sectional Study in China. Vaccines.

[191] Michel J., Sauter T.C., Tanner M. (2021). Vaccine hesitancy and its determinants—A way forward?. J. Glob. Health Econ. Policy.

[192] de Kiev L.C., Danny A.S. (2022). Vaccine Hesitancy in the Post-COVID-19 Era: An Interdisciplinary Approach for A Trust-and-Risk Paradigm with Governmental and Intergovernmental Implication. Intergov. Res. Policy J..

