# Supplementary material for: Quantitative Synthesis of Factors Associated with COVID-19 Vaccine Acceptance and Vaccine Hesitancy in 185 Countries
Source: Vaccines (Basel). 2023 Dec 28;12(1):34. doi: 10.3390/vaccines12010034 (PMC10818751; doi:10.3390/vaccines12010034)
Supplement: Supplementary file 1 [file vaccines-12-00034-s001.zip › vaccines-2779668-supplementary.pdf]

Supplementary material

Table S1: Factors associated with COVID-19 vaccine acceptance that were selected and analyzed

Factors identified in the cross-sectional VA studies selected for our analysis	Factors grouped using the Health Belief Model (demographic, psychosocial, and structural variables) for further analysis in this study.
• 18 – 30 years<br>• 30 – 40 years<br>• 40 – 50 years<br>• 51 – 60 years	Younger people
• Above 60 years	Older people
• Male	Male
• Female	Female
• Rural	Rural
• Urban	Urban
• No education<br>• Primary education<br>• Secondary education	Low education
• Professional training<br>• University level<br>• Postgraduate level	High education
• Smoker	Smoker
• Non – smoker	Non – smoker
• Self-protection<br>• Protect others	Protection
• Not married<br>• Single	Single
• Married<br>• Living with spouse	Married
• Living with children<br>• Living with elderly	Living with relatives
• Lower income	Lower income earner
• Higher income	Higher income earner
• Medical students<br>• Nurses<br>• Midwife<br>• Laboratory technicians<br>• Medical doctors<br>• Community health workers	Healthcare worker
• Other vaccines for childhood<br>• Other vaccines for adults<br>• Influenza vaccine	Influenza/Other vaccines
• student	Student
• private sector	Private sector
• Christians	Christians
• Muslims	Muslims
• No adverse effect	Safety

• No side-effects<br>• Safety	
• High risk of infection	High risk of infection
• Effectiveness<br>• Efficacy<br>• Trust COVID-19 vaccines<br>• Trust manufacturer	Effectiveness
• Scared of infection<br>• Prior infection<br>• Knows someone who has been infected<br>• Tested positive for COVID-19<br>• COVID-19 is dangerous and kills<br>• Compliance with protective measure	Fear of infection
• Chronic disease<br>• Poor health<br>• Comorbidities	Comorbidities
• No previous infection<br>• Good health	Good health
• Reliable information<br>• social responsibility<br>• Trust Ministry of Health<br>• Trust government<br>• Vaccine recommended by healthcare workers<br>• Believe in community/public leaders<br>• Trust<br>• Vaccines are important	Trust
• Frequent traveller	Frequent traveller
• Forced vaccination<br>• Mandatory vaccination<br>• Compulsory by employer<br>• Compulsory by gov't	Mandatory vaccination

Table S2: Factors associated with COVID-19 vaccine hesitancy that were selected and analyzed in this study

Factors identified in V-H studies	Factors grouped using the Health Belief Model (demographic, psychosocial, and structural variables) for further analysis in this study.
• 18 – 30 years<br>• 30 – 40 years<br>• 40 – 50 years<br>• 51 – 60 years	Younger people
• Above 60 years	Older people
• Male	Male
• Female	Female
• transgender	Transgender
• Rural	Rural

• Urban	Urban
• No education<br>• Primary education<br>• Secondary education	Low education
• Professional training<br>• University level<br>• Postgraduate level	High education
• Smoker	Smoker
• Non – smoker	Non – smoker
• Not married<br>• Single	Single
• Married<br>• Living with spouse	Married
• Lower income	Lower income earner
• Higher income	Higher income earner
• Medical students<br>• Nurses<br>• Midwife<br>• Laboratory technicians<br>• Medical doctors<br>• Community health workers	Healthcare worker
• No other vaccines<br>• No Influenza vaccine	No influenza/Other vaccines
• Christians	Christians
• Muslims	Muslims
• Black	Black
• White	White
• Chronic diseases<br>• Poor health<br>• Comorbidities	Comorbidities
• Pregnancy	Pregnancy
• Breastfeeding	Breastfeeding
• adverse effect<br>• side-effects<br>• unsafe<br>• safety	Safety
• Vaccines do not work<br>• I will not get infected<br>• Not concerned<br>• COVID-19 is not dangerous<br>• COVID-19 is Whiteman's disease<br>• COVID-19 does not exist<br>• Good health<br>• Getting infected is better protection<br>• Complacency	Complacency
• traditional herbs/alternative med	traditional herbs/alternative med
• ineffective	Ineffectiveness

• non-efficacious	
• Prior infection	Prior infection
• mistrust vaccine<br>• conspiracy<br>• misinformation/social media<br>• mistrust health system<br>• mistrust manufacturer<br>• mistrust scientist	Mistrust vaccine
• misinformation<br>• lack of information<br>• social media<br>• anti-vaxxers	Lack of information
• other vaccines	Other vaccines
• Mistrust ruling party<br>• Mistrust gov't<br>• Mistrust opposition party	Political mistrust
